# A diabetic mouse model of early stages of *Clostridium perfringens*-gas gangrene

**DOI:** 10.1016/j.anaerobe.2026.103050

**Published:** 2026-05-04

**Authors:** Francisco A. Uzal, Jihong Li, Juliann Beingesser, Jessica Banuelos, Mauricio A. Navarro, Bruce A. McClane

**Affiliations:** a California Animal Health and Food Safety Laboratory System, School of Veterinary Medicine, University of California-Davis, San Bernardino, CA, USA; b Department of Microbiology and Molecular Genetics, University of Pittsburgh School of Medicine, Pittsburgh, PA, USA; c California Animal Health and Food Safety Laboratory System, School of Veterinary Medicine, University of California-Davis, Turlock, CA, USA

## Abstract

**Introduction:**

Since *Clostridium perfringens* gas gangrene is strongly associated with human diabetes, this study compared gas gangrene severity in *db/db* (a model for type 2 diabetes) *vs* wild-type (WT) mice after a 4 h *C. perfringens* challenge.

**Material and methods:**

Before *C. perfringens* challenge, glucose and branched chain amino acids (BCAA) concentrations were significantly higher in *db/db* vs. WT mice. All mice were then inoculated intramuscularly in, i) the left thigh with DPBS containing 10^6^, 10^7^ or 10^8^ washed cells of *C. perfringens* type A strain ATCC3624 and ii) the right hind thigh with DPBS. After 4 h, mice were euthanized and samples of muscle and subcutaneous tissue from those thighs were collected for anaerobic culture or histology examination.

**Results:**

Gross and microscopic lesions in the left thigh were influenced by bacterial dose. With 10^7^ or 10^8^ bacterial challenges, the *db/db* mice showed more severe gross and microscopic lesions than did WT mice. No gross or microscopic lesions were observed in the right thigh of any mice. No significant differences were noted in *C. perfringens* numbers recovered from the left thigh of *db/db vs* WT mice at any challenge inoculum. After a 2 h challenge, RT-qPCR detected more expression of genes encoding alpha toxin and perfringolysin O in the *db/db* than in WT mice.

**Discussion:**

This study supports the usefulness of *db/db* mice to study *C. perfringens* gas gangrene pathogenesis in diabetics. It also suggests that higher BCAA and glucose levels and/or stronger upregulation of toxin production in *db/db* mice may be factors that help cause the development of more severe lesions during the early stages of gas gangrene in the *db/db* mice.

## 1. Introduction

*Clostridium perfringens* is an anaerobic, gram-positive, spore-forming rod that causes several human and animal diseases. Among those are soft tissue infections such as gas gangrene, also known as clostridial myonecrosis [1]. This bacterium is classified into seven types (A to G) based upon carriage of genes encoding six toxins, i.e. alpha (CPA), beta, epsilon, iota, enterotoxin, and necrotic enteritis B-like toxin [2].

*C. perfringens* type A, which encodes CPA but none of the other typing toxins, is responsible for 80–90% of the ~1000 traumatic human gas gangrene cases that occur annually in the USA (https://emedicine.medscape.com/article/214992-overview?form=fpf). CPA, which has phospholipase C and sphingomyelinase activities, is the main *C. perfringens* virulence factor for producing gas gangrene [3,4]. However, perfringolysin O (PFO), a pore-forming toxin, also works synergistically with CPA during development of this disease [5]. CPA and PFO restrict extravasation of inflammatory cells, with the result that the site of infection has a less prominent inflammatory cell infiltrate [6–8]. These toxins also induce upregulation of adhesion molecules on the surface of inflammatory cells, which promotes intravascular cell aggregation leading to thrombosis, thereby reducing tissue redox conditions to facilitate *C. perfringens* growth [4,8–11]. Recently, PFO and CPA were also shown to promote *C. perfringens* growth *in vitro* using a differentiated mouse muscle cell line, suggesting that these toxins similarly release nutrients from muscle cells when *C. perfringens* grows during gas gangrene [12].

*C. perfringens* gas gangrene typically initiates when traumatic injuries facilitate the entry of spores or vegetative cells of this bacterium deep into tissue, including muscle [13]. Clinically, gas gangrene is characterized by fever, pain, local swelling and skin discoloration, usually progressing to toxemia, sepsis, shock and death [14]. Lesions include necrosis of skeletal muscle, and edema and emphysema of skeletal muscle and subcutaneous tissue. Even with therapy, gas gangrene still has an overall 20–40% mortality [15]. Therapy for gas gangrene often involves limb amputation, which not only results in amputees being debilitated for life but also leaves them prone to future medical problems since the 5-year post-amputation survival rate is only ~20% [16].

Approximately 80–90% of traumatic wounds are contaminated with *C. perfringens* [17]. However, only ~0.8% of those contaminated wounds develop into gas gangrene [17]. This clinical observation highlights the importance of host factors for the development of clostridial myonecrosis. One well-established major host risk factor for gas gangrene is diabetes mellitus, particularly type 2 [15,16,18–21]. Diabetes affects ~600 million people worldwide and is rapidly increasing in incidence due to factors such as rising obesity rates and sedentary lifestyles (https://diabetesatlas.org). Recent clinical surveys showed that 80% of gas gangrene cases occurred in diabetics [20] and, even with treatment, the fatality rate for gas gangrene in diabetics is much higher (67%) compared to an overall 20–40% mortality rate for non-diabetic patients [15].

The association between diabetes and gas gangrene is considered multifactorial and is thought to involve host factors such as diabetics having poor vascular flow, which causes tissue hypoxia that helps *C. perfringens* grow in muscle, poor wound healing, and a suppressed immune system [15,21]. Another possible host contributor to the association between gas gangrene and diabetes is not generally considered but could involve the numerous metabolic disturbances in diabetics. For example, diabetics have much higher blood levels of glucose and branched chain amino acids (BCAA) than nondiabetic people [5,22, https://diabetes.org/living-with-diabetes/treatment-care/hyperglycemia]. Increasing evidence suggests that high BCAA and glucose levels in type 2 diabetics may be linked, e.g., elevated BCAA levels may impact glucose metabolism in diabetics and suppress wound healing and immune responses [8,13,22–24].

The elevated glucose and BCAA levels in type 2 diabetics might also impact *C. perfringens* growth or toxin production since, i) this bacterium can use glucose for growth [25] and ii) *C. perfringens* lacks the ability to synthesize BCAAs [26,27] so it must obtain BCAAs from the host during infections such as gas gangrene. Supporting their importance for *C. perfringens* growth during disease, it has been known since the 1940s that BCAAs are essential for growth of this bacterium *in vitro* [28,29]. Gas gangrene in diabetics can have a polymicrobial nature, so the high glucose or BCAA levels in diabetics might also broadly influence interactions of those bacteria with *C. perfringens* [30].

Small animal models are useful to study the complex pathogenesis of gas gangrene. In particular, a widely used BALB/c mouse hind-limb model has provided substantial insights into gas gangrene pathogenesis [3,5,9,10,28,31]. However, no animal model has yet been applied to probe the strong association between *C. perfringens* gas gangrene and diabetes. This study reports the use of *db/db* mice as a model and to begin exploring the linkage between diabetes and *C. perfringens* gas gangrene, i.e., can we demonstrate more severity in these diabetic *vs* normal mice?

## 2. Results

### Glucose and BCAA measurement.

**Fig. 1.**
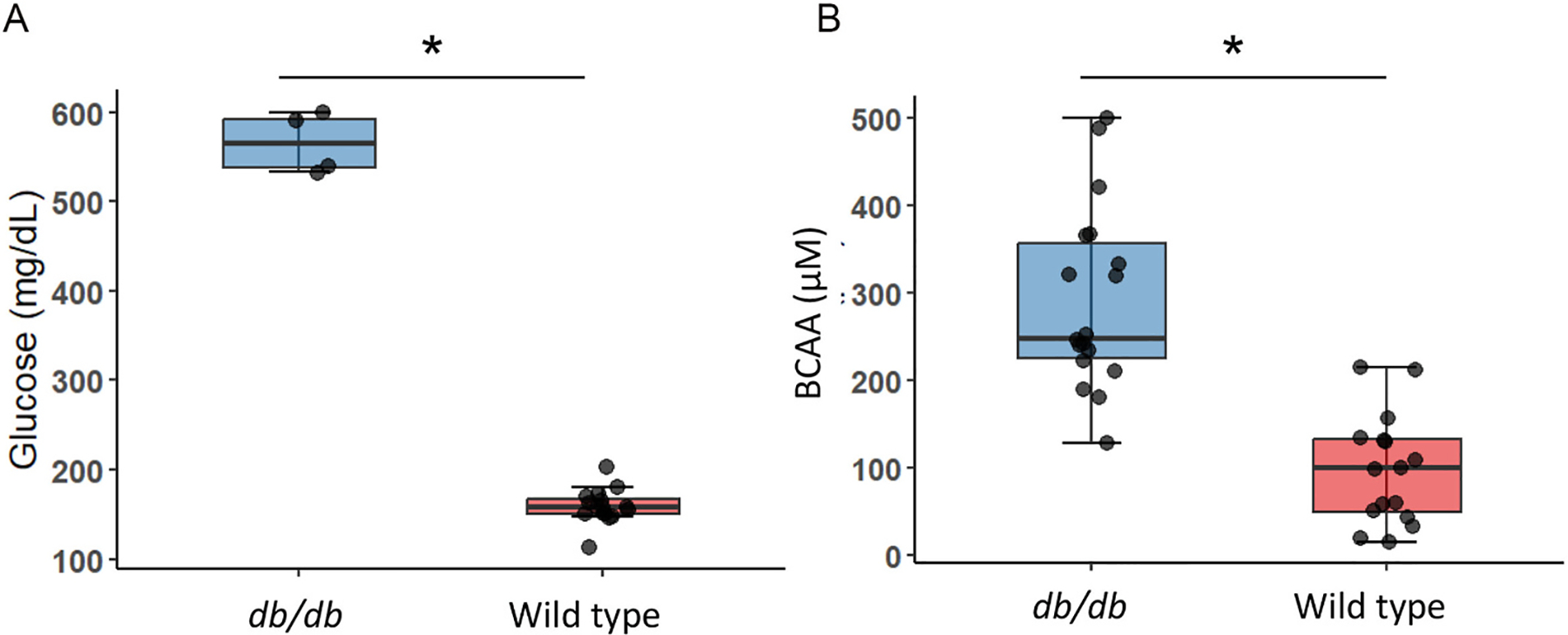
Comparison of glucose and BCAA levels in ~5-week-old *db/db* vs. normal wild type (WT) mice. (A) The average glucose concentration was significantly higher in *db/db* mice than in WT mice. Glucose levels were measured in 14 or 16, respectively *db/db* or control mice prior to *C. perfringens* challenge. Only glucose values < 600 mg/dl are shown in Panel A and were used for statistical analyses since higher values in most *db/db* mice were off-scale for our glucose monitor. (B) BCAA blood concentration was significantly higher in 18 db/db mice than in 16 WT mice. Data are represented as boxplots (median ± interquartile range) with individual data points shown. Statistical significance was determined by independent *t*-test. The asterisks indicate significant differences (P < 0.05). Equal numbers of each sex are included.

Many previous studies [32–35] have reported that *db/db* mice develop elevated glucose and BCAA levels over time. To confirm that result for our six-week-old mice, and compare those values against matching wild type (WT) control mice, we measured BCAA and glucose levels immediately prior to the start of our experiments, i.e., before challenge with *C. perfringens*. Most (14/18, or ~78%) *db/db* mice had blood glucose concentrations exceeding the 600 mg/dl limit of our glucose monitor. Therefore, for calculation purposes, we considered only those mice whose blood glucose levels were measurable, i.e., ≤600 mg/dl. Even with that conservative caveat, the average glucose concentration was still consistently higher in *db/db* mice than in WT mice (Fig. 1A). Similarly, BCAA blood concentration was significantly higher in *db/db* mice than in WT mice (Fig. 1B).

### Clinical and gross changes.

**Fig. 2.**
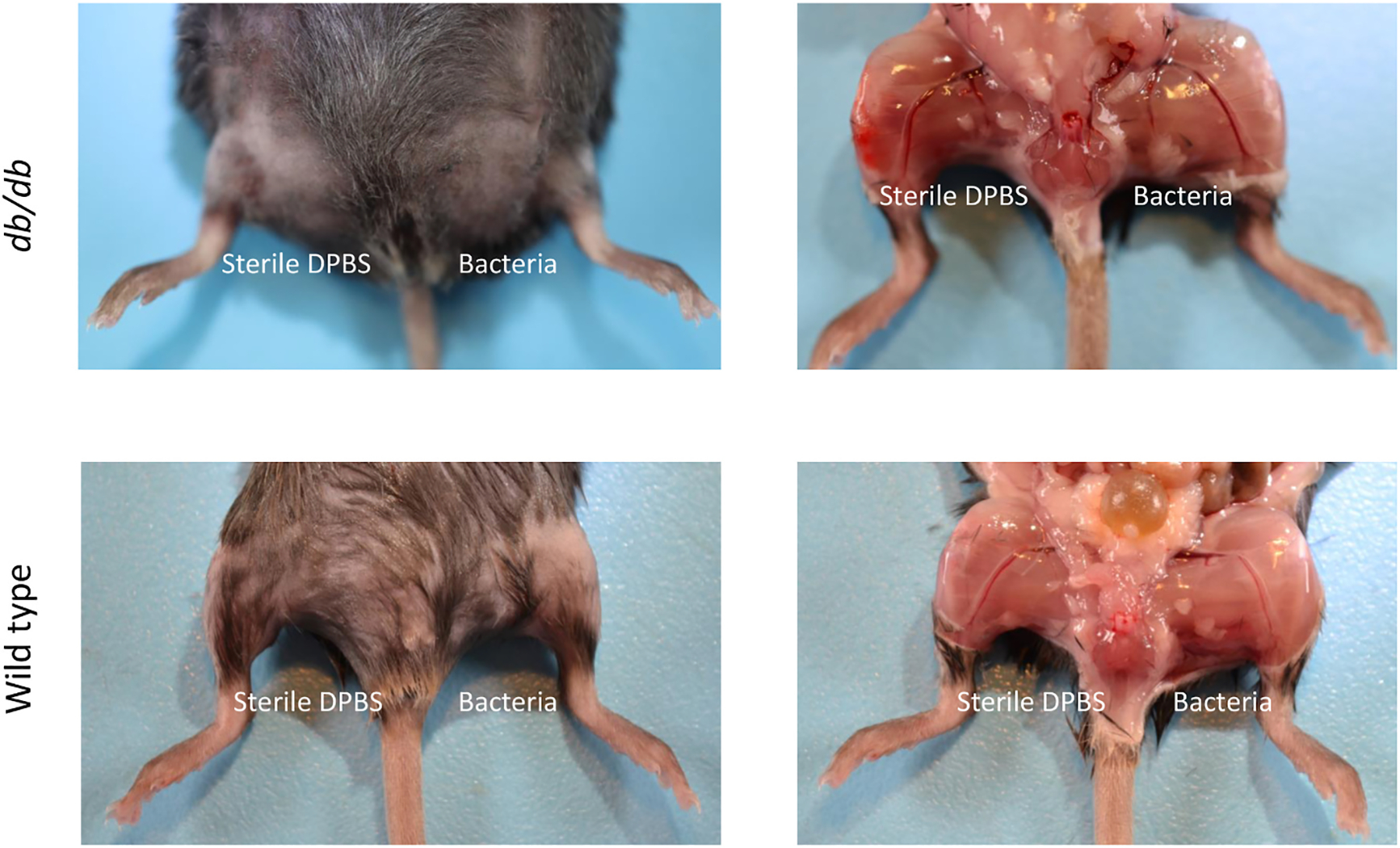
Post-mortem examination of mice. Gross lesions were present only in the left thigh of both *db/db* and WT mice, which had been challenged for 4 h with *C. perfringens* type A strain ATCC3624. Those lesions included swelling and dark red discoloration of the skin, subcutaneous tissue and skeletal muscle. There was also subcutaneous and thigh muscle edema and hemorrhage. The lesions were more severe in the left thigh of *db/db* than in the left thigh of WT mice. No gross lesions were observed in the right thigh that received an injection of sterile DPBS. Thirty *db/db* (15 male and 15 female) and 30 WT (15 male and 15 female) C57BL/6J Jackson mice were used.

Starting ~1 h after inoculation, all mice challenged with *C. perfringens* developed clinical abnormalities that included limping, and swollen and hot legs. In all of those animals, the clinical signs grew progressively more severe throughout the 4 h incubation period. Post-mortem examination revealed gross lesions only in the left thighs, which had been challenged with *C. perfringens* type A strain ATCC3624 (Fig. 2). Those lesions included swelling and dark red discoloration of the skin, subcutaneous tissue and skeletal muscle. Subcutaneous and thigh muscle edema and hemorrhage were also present.

The extent of these changes was dependent upon the dose of the ATCC3624 challenge. For both *db/db* and WT mice, the 10^8^ ATCC3624 challenge caused the most severe lesions, while mice inoculated with 10^7^ or 10^6^ CFU of this microorganism had progressively less severe lesions. No gross lesions were observed in the right hind thigh of any mice, which had been challenged with sterile DPBS. For each *C. perfringens* challenge dose, the clinical signs were more severe in *db/db* animals than in WT mice.

### Histopathology.

**Fig. 3.**
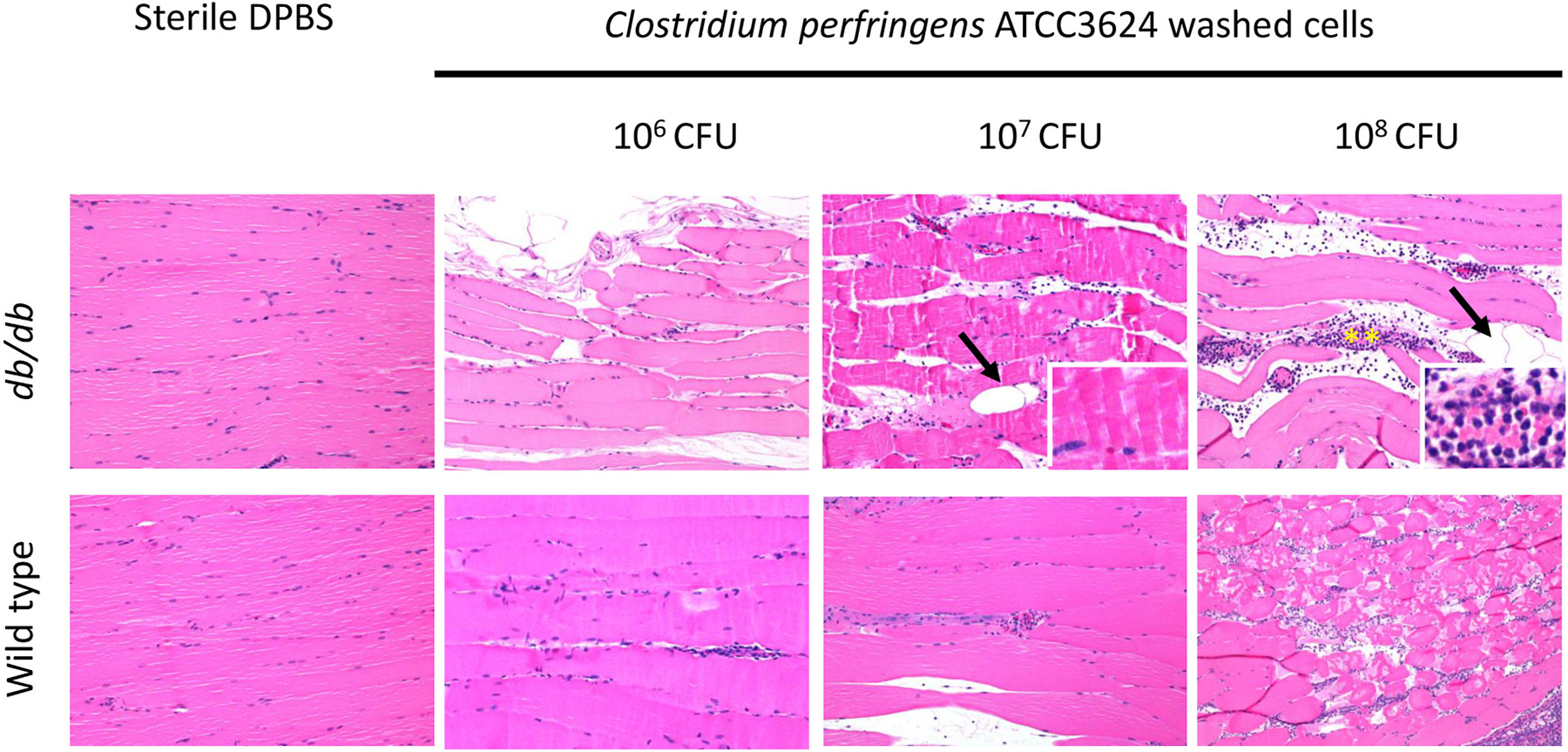
Microscopic lesions in muscle of mice challenged with ATCC3624 (right three panels) or DPBS (left panel). For both *db/db* and WT mice, microscopic lesions were observed only in the left thigh which was challenged for 4 h with ATCC3624. These lesions consisted of edema, hemorrhage, emphysema (arrows), infiltration of neutrophils (asterisks) and fewer lymphocytes, plasma cells and macrophages, and changes in muscle fibers, including loss of cytoplasm and striations, vacuolation, swelling and presence of hypercontraction bands. The lesions were progressively more severe in mice challenged with 10^6^, 10^7^ and 10^8^ CFU of *Clostridium perfringens*. No significant microscopic lesions were observed in the muscle of the right thigh of either group of animals, which was injected with sterile DPBS. Inset in figure of *db/db* mice inoculated with 10^7^ CFU of *Clostridium perfringens* shows higher magnification of hypercontraction bands in muscle of the left thigh. Inset in figure of *db/db* mice inoculated with 10^8^ CFU of *Clostridium perfringens* shows higher magnification of inflammatory cells, mostly neutrophils in the left thigh.

**Fig. 4.**
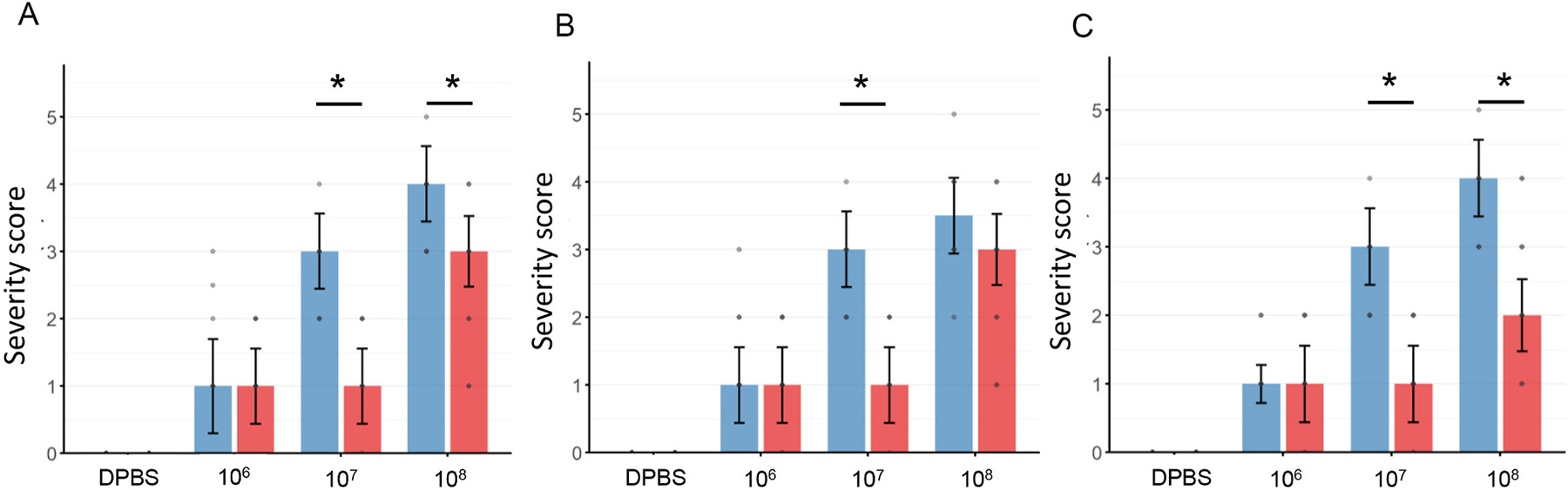
Quantitative scoring of microscopic lesions. (A) The overall severity score of microscopic lesions was higher for *db/db* than for WT mice and this difference reached statistical significance for mice challenged for 4 h with either a 10^7^ or 10^8^ inoculum of *C. perfringens* type A strain ATCC3624. The overall score was calculated based on the severity score of inflammation and necrosis. (B and C) Inflammation and necrosis were significantly more severe for mice challenged with either a 10^7^ or 10^8^ inoculum of *C. perfringens* type A strain ATCC3624. Median histological scores (0–5). Error bars represent 95% confidence interval (Notch formula). The asterisks indicate significant differences (P < 0.05). Slides from twenty four *db/db* (12 male and 12 female) and 25 WT (13 male and 12 female) C57BL/6J Jackson mice were used.

After a 4 h challenge, microscopic lesions were observed only in the left thigh of all mice. These lesions consisted of edema, hemorrhage, infiltration of neutrophils and fewer lymphocytes, plasma cells and macrophages, and changes in muscle fibers, including loss of cytoplasm and striations, vacuolation, swelling and presence of hypercontraction bands (Figs. 3 and 4). The severity score of these microscopic lesions was higher for *db/db* than for WT mice and this difference reached statistical significance for mice challenged with either a 10^7^ or 10^8^ inoculum of *C. perfringens* type A strain ATCC3624 (Fig. 4A). In those mice there were also significant statistical differences in the severity of inflammation (with a 10^7^ challenge) and necrosis (with either a 10^7^ or 10^8^ challenge) between *db/db* and WT mice (Fig. 4B and C).

### Presence of *Clostridium perfringens* in thigh muscle of the challenged mice.

**Fig. 5.**
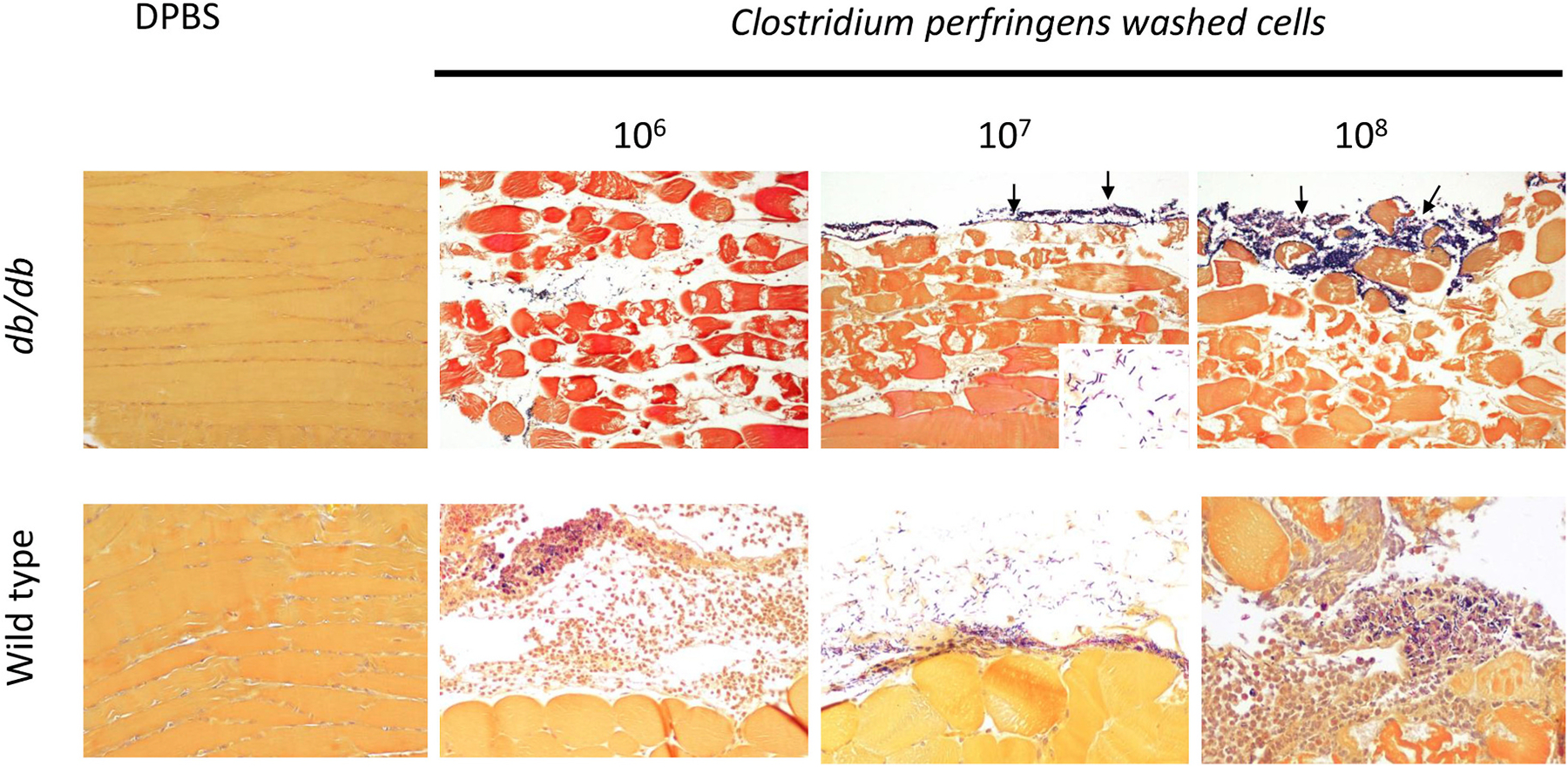
Presence of *Clostridium perfringens* in mouse muscle challenged for 4 h with ATCC3624 (right three panels) or DPBS (left panel). Myriad gram-positive, non-sporulated rods were observed associated with the lesions in the left thigh muscle and subcutaneous tissue of mice that had been challenged with *C. perfringens* type A strain ATCC3624. The number of gram-positive bacilli was higher in mice inoculated with higher doses of this microorganism. No bacteria were present in right hind thigh muscle challenged with DPBS. Thirty *db/db* (15 male and 15 female) and 30 WT (15 male and 15 female) C57BL/6J Jackson mice were used.

**Fig. 6.**
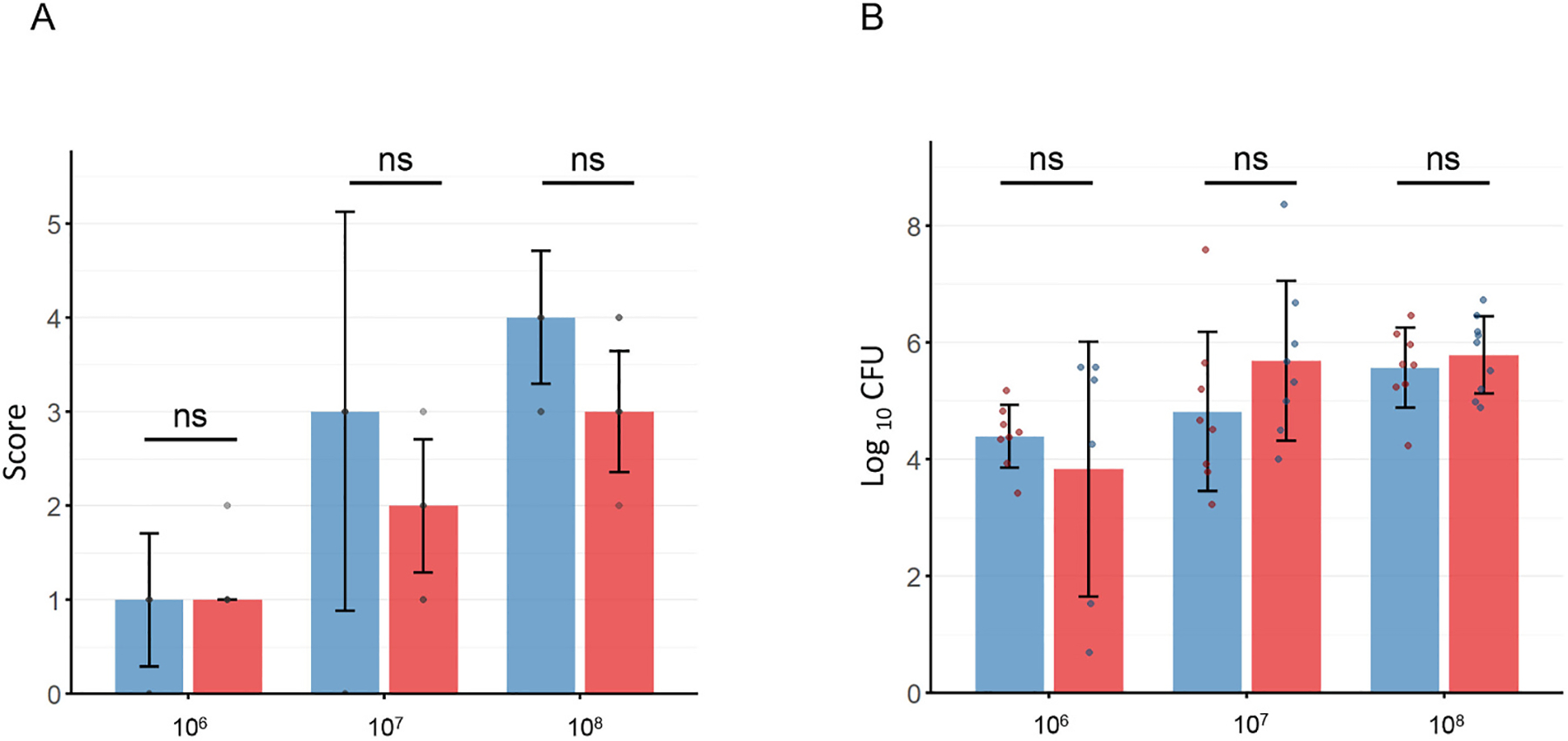
Quantitative analyses of ATCC3624 presence in challenged muscle. (A) No significant differences in the number of bacteria were observed between *db/db* and WT mice when Gram-stained sections of muscle and subcutaneous tissue were examined. However, in both groups of mice, significantly (P < 0.05) more bacteria were present with higher doses of inoculum. Median histological scores (0–5). Error bars represent 95% confidence interval (Notch formula). (B) Skeletal muscle and subcutaneous tissue were aseptically collected from the left and right hind thighs of all mice and those samples were then plated on *C. perfringens* selective agar to culture overnight for CFU calculations. No significant differences in the number of viable *C. perfringens* CFU recovered from the left thigh of *db/db* vs. wild type mice when challenged for 4 h with the same *C. perfringens* inoculum. Bacterial CFU values (mean ± SD of Log_10_ CFU) were compared using Welch's *t*-test methodology. Twenty four *db/db* (12 male and 12 female) and 23 WT (12 male and 11 female) C57BL/6J Jackson mice were used.

Two experiments were then performed to evaluate the presence of *C. perfringens* cells in muscle of the challenged *db/db* vs WT mice. First, the formalin-fixed, paraffin-embedded tissues were stained with Gram and examined by microscopy. Myriad gram-positive, non-sporulated rods were associated with the lesions in the left thigh muscle and subcutaneous tissue of all mice but not in their right hind thigh muscle or subcutaneous tissue (Fig. 5). For both the *db/db* and WT mice, the abundance of those bacteria in the left thigh muscles increased as the challenge dose became larger (Fig. 6A). However, this approach did not detect any significant differences in *C. perfringens* abundance in the left thigh muscles between *db/db* and WT mice challenged with the same ATCC3624 inoculum. Confirmation that these rods were *C. perfringens* was obtained by *C. perfringens* immunohistochemistry, for which these microorganisms were positive (not shown).

A second approach specifically quantifying viable *C. perfringens* in these tissues was then applied, whereby skeletal muscle and subcutaneous tissue was aseptically collected from the left and right hind thighs of all mice and those samples were then plated on *C. perfringens* selective agar to culture overnight for CFU calculations. This second approach detected no significant differences in the number of viable *C. perfringens* CFU recovered from the left thigh of *db/db* vs. WT mice when challenged for 4 h with the same *C. perfringens* inoculum (Fig. 6B). No *C. perfringens* were isolated from the right hind thigh of any *db/*db or WT mice (controls).

As a specificity control, five randomly selected colonies recovered from the left thighs of *db/db* and WT mice were screened by PCR for the presence of the alpha toxin (*cpa)* gene to confirm their identity as *C. perfringens*. All colonies tested positive for *cpa* gene carriage (data not shown).

### Isolation of *Clostridium perfringens* RNA from muscle of infected mice and RT-qPCR analysis of toxin gene expression.

Since the total or viable bacterial loads did not significantly differ between *db/db* and WT mice challenged with the same ATCC3624 inoculum, it was possible that a mechanism by which this type A strain ATCC3624 caused more damage in the muscle of diabetic mice *vs* WT mice might involve the heightened presence in muscle of CPA and PFO, which are both important toxins for gas gangrene [3,5].

**Fig. 7.**
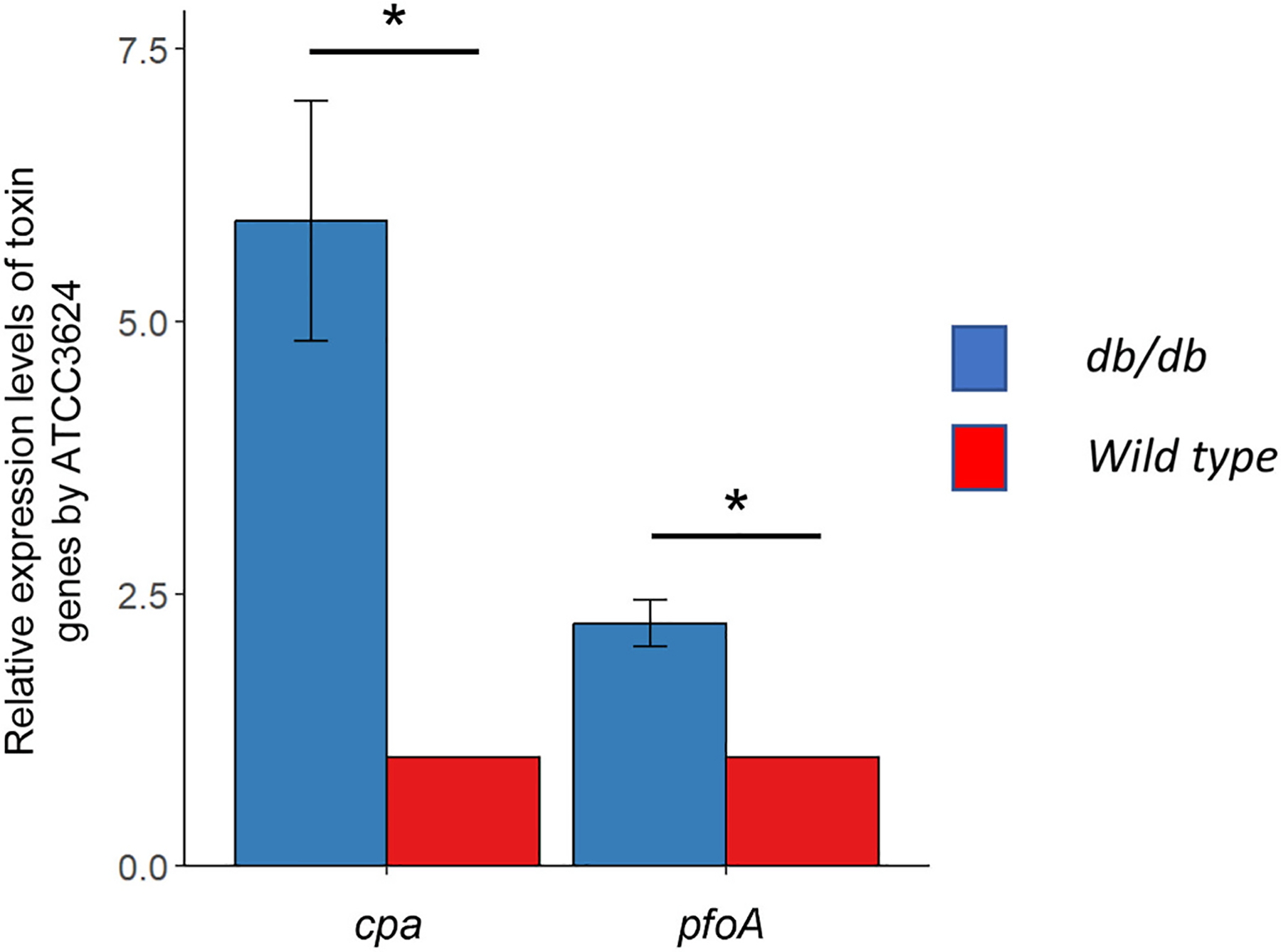
RT-qPCR comparison of *cpa* and *pfoA* expression levels between *db/db* vs WT mice. Within 2 h of a 10^7^ ATCC3624 challenge into the left thigh of these mice, there was a significant increase in expression levels of both toxin genes in *db/db vs* WT mice, but particularly for the *cpa* gene. Data are represented as bars with mean ± standard error. Statistical significance was determined by independent *t*-test. Results shown are average of muscle samples from 1 male and 2 female mice, each repeated twice. The asterisks indicate significant differences (P < 0.05).

Attempts to quantify PFO and CPA levels in infected tissues by ELISA were not reliable because they were too near the assay sensitivity limits, likely due to issues such as binding of these toxins to muscle cells. Therefore, as used in a previous study [36] examining ATCC3624 infection of differentiated mouse C2C12 muscle cells, the current study performed RT-qPCR to compare *cpa* and *pfoA* expression levels in mouse muscle tissue after inoculation of type A strain ATCC3624 into the thigh muscle of *db/db* mice *vs*. WT mice. The results (Fig. 7) indicated that, within 2 h of a 10^7^ ATCC3624 challenge into the left thigh of these mice, there was a significant increase in expression levels of both toxin genes, but particularly the *cpa* gene, in *db/db vs* WT mice. This finding suggests that, early during gas gangrene, CPA and PFO production may be higher in these diabetic mice compared to wild-type mice.

No sex-related differences in results were noted for either *db/db* or WT mice in any of the experiments described above.

## 3. Materials and methods

### Bacteria, media and reagents.

This study used *C. perfringens* type A strain ATCC3624, which was purchased from the American Type Culture Collection (ATCC). The following media were employed: Fluid thioglycolate medium (FTG) (Difco Laboratories); TY broth (3% tryptic soy broth [Becton-Dickinson], 1% yeast extract [Becton Dickinson], and 0.1% sodium thioglycolate [Sigma-Aldrich]). Tryptose-sulfite-cycloserine (TSC) agar plates made of SFP agar base (Becton, Dickinson) with 0.04% D-cycloserine (Sigma-Aldrich) were used for selective isolation of *C. perfringens* from challenged skeletal muscle of mice. All other chemical reagents used in this study were purchased from Fisher Scientific, Sigma Aldrich or Bio-Rad. Each experiment was performed using 3 groups of mice (3 separately prepared inocula) and each mouse was evaluated independently.

### Inocula preparation.

For production of inocula, a 0.4 ml aliquot of a cooked meat stock culture of ATCC3624 was transferred into 20 ml of FTG and incubated overnight at 37 °C. That culture was diluted 1:10 with fresh FTG and 10 ml of that diluted culture was inoculated into 250 ml of TY medium. After 5 h at 37 °C, 2, 20 or 200 ml of the TY culture were removed for optical density measurement at 600 nm (OD_600_) using a Bio-Rad Smart Spectrophotometer. These aliquots were centrifuged and the pellet was suspended in sterile DPBS to reach concentrations of 10^6^, 10^7^ and 10^8^ CFU in 50 μl final volume.

### Animals.

Thirty diabetic *db/db* (15 male and 15 female) C57BL/6J mice (Jackson Laboratories) were used. These mice are homozygous for a diabetes spontaneous mutation (*Lepr*^*db*^). These *db/db* mice become obese around three to four weeks of age and have elevations of plasma insulin beginning at 10 to 14 days of age, and of blood glucose at 4 to 8 weeks of age. They are polyphagic, polydipsic and polyuric (https://www.jax.org/strain/000642). Another 30 (15 male and 15 female) normal wild type (WT) C57BL/6J Jackson mice were used for matching nondiabetic animal comparisons. All the mice were received at ~5 weeks of age and were held at the laboratory for ~1 week before they were used for the experiments. They were offered water and pelleted food ad-libitum and subjected to a regular light/darkness cycle in a temperature and humidity-controlled environment.

### Mouse model of gas gangrene.

On the day of each experiment, blood was collected from the external saphenous vein and blood from 18 animals was tested for glucose levels using a commercial blood glucose monitor (VQPET H blood glucose monitoring system, Germaine Laboratories, San Antonio, TX) and BCAA levels were evaluated using a colorimetric assay (Branched amino acid assay kit, Cell Biolabs Inc., San Diego, CA) following the manufacturer's instructions.

The mice were then divided into three groups (groups 1, 2 and 3) of 20 animals each (10 db/db and 10 WT per group, with each group comprised of equal numbers of male and female mice). Each group was then inoculated intramuscularly into the left thigh with 10^6^, 10^7^ or 10^8^, respectively, washed cells of *C. perfringens* type A strain ATCC3624 diluted in sterile DPBS at a final volume of 50 μl. Three separate cultures of ATCC3624 were used for these experiments and no variations were noted using those different cultures. Fifty microliters of sterile DPBS were also inoculated intramuscularly in the right hind thigh of all animals. The mice were examined clinically before inoculation and then periodically for 4 h after inoculation, when they were euthanized by cervical dislocation and a full necropsy was performed. All experiments involving mice were reviewed and approved by the University of California, Davis, Institutional Animal Care and Use Committee (protocol 20513).

### Histopathology.

Samples of muscle and subcutaneous tissue were collected, fixed in 10% buffered formalin pH 7.2 for 24 h, and processed routinely for the production of 4μ thick, hematoxylin and eosin (HE)-, or Gram-, stained sections. HE-stained histologic slides from four mice per treatment were evaluated by a board-certified pathologist in a blinded fashion. A semiquantitative score of lesion severity was assigned to each section using a numerical scale from 0 to 5. Each score was calculated based on the average of the examination of 5 different 200X microscopic fields. For HE-stained sections, a score of 0 was assigned when no lesions were observed; while scores of 1, 2, 3, 4 and 5 were assigned when the lesions were progressively more severe. For this score, the following parameters were used: changes in the interstitium (edema, hemorrhage, presence of inflammatory cells), and changes in muscle fibers (loss, vacuolation and swelling of cytoplasm, hypercontraction bands and nuclear pyknosis). In particular, the presence and quantity of inflammatory cells, which is considered the gold standard to establish inflammation in tissues, was used to determine the severity of inflammation. For necrosis, the following parameters were considered: cytoplasmic acidophilia, loss of striations, loss of cytoplasm, vacuolation of cytoplasm and presence of hypercontraction bands. For Gram-stained sections, a score of 0 to 5 was also used, with a score of 0 assigned when no bacteria were observed, and scores of 1, 2, 3, 4 and 5 when the number of bacteria became progressively larger.

### *Clostridium perfringens* immunohistochemistry.

Additional sections of skeletal muscle were processed by an immunohistochemistry technique for *C. perfringens,* as previously described [37] using a Dako EnVision kit (Dako, Carpenteria, CA) following the instructions of the manufacturer. A rabbit polyclonal *C. perfringens* antibody (GenWay Bio, San Diego, CA) was used as a primary reagent, a peroxidase labelled polymer conjugated to goat anti-mouse or goat anti-rabbit as secondary reagent and 3,3′-diaminobenzidine as chromogen.

Negative controls included sections of skeletal muscle from mice that had not been challenged with *C. perfringens* or sections from mice challenged with this microorganism but incubated with normal rabbit serum instead of the specific antibodies. Sections of colon from a goat which had been challenged with *C. perfringens* were used as a positive control.

### Recovery of *C*. *perfringens* from challenged tissues.

Samples of muscle and subcutaneous tissue from both hind thighs were aseptically collected, weighed, macerated and resuspended in DPBS. Serial dilutions (10^−1^ to 10^−8^) were prepared in DPBS before plating aliquots onto TSC agar plates selective for *C. perfringens*, followed by overnight incubation under anaerobic conditions at 37 °C. The number of black colonies, corresponding to *C. perfringens* [38], was recorded to calculate the number of CFU per gram of muscle. Five of those colonies from *db/db* mice and five from WT mice, were tested by PCR for the *cpa* gene, to confirm that they were *C. perfringens,* as previously described [4].

### Isolation of *C. perfringens* RNA from muscle of infected mice muscle and RT-qPCR analysis for toxin gene expression.

After infection of *db/db* or wild-type mice with 10^7^ CFU of ATCC3624 for 2 h, samples were collected from infected muscle. All samples were frozen at −80 °C before RNA extraction. The muscle samples (0.2 to 0.6 g) were homogenized with 1 ml of TE buffer (Sigma). A 100 μl aliquot of a 20 mg/ml proteinase K solution (Fisher Scientific) was added to each sample for 10 min at 55 °C. The samples were centrifuged at 3500×*g* for 20 min. The pellets, which contained the bacterial cells, were resuspended in 1 ml TE buffer with 0.1% Triton X-100 and then centrifuged at 5000×*g* for 5 min. The resultant pellets were washed 3 to 4 times with TE buffer with 0.1 % Triton X-100 to enrich for *C. perfringens* cells. This method was modified from a previous study [39].

RNA was extracted from those samples using the saturated phenol method and purified by TRIzol and chloroform (Life technologies and Sigma), as described previously [40]. The extracted RNA was then quantified by measuring absorbance at 260 nm with a Bio-Rad Smart Spectrophotometer. To perform RT-qPCR for the *plc* or *pfoA* genes, an aliquot (1 μg) of purified RNA was then used for first strand cDNA synthesis with the Thermo Scientific Maxima First Strand cDNA synthesis kit. Reaction mixtures were incubated in a thermal cycler for 10 min at 25 °C, 30 min at 50 °C and 5 min at 85 °C to allow cDNA synthesis. For RT-qPCR, cDNA samples were diluted two-fold. To perform RT-qPCR, results were normalized to the reference gene *recA* and fold changes were then calculated using the 2^−*ΔΔC*^method [41]. Primers used for this experiment were the same as listed previously [12].

#### 3.1. Statistical analyses

All statistical evaluations were performed using RStudio 2026.01.2. For continuous variables (glucose and BCAA levels, and relative gene expression), normality was assessed using the Shapiro-wilk test, and homogeneity of variances was evaluated using Levene's test. Differences were compared using an independent samples *t*-test, and the results are expressed as mean ± standard error of the mean (SEM). Histological scores were treated as ordinal data; differences in the distribution of scores between groups were analyzed using the non-parametric Mann-Whitney *U* test. Histological results are presented as median values with interquartile ranges (IQR). For all analyses, a p-value <0.05 was considered statistically significant. For microbial load, raw CFU values were log_10_ transofrmed to normalize the distribution and stabilize variance. Normality was confirmed for each group independently using the Shapiro-Wilk test (p > 0.05) and visual inspection of Q-Q plots. Differences in the man log-microbial load between the diabetic and control groups were evaluated using a two-tailed independent samples *t*-test (Welch's *t*-test). CFU data are presented as mean ± standard deviation (SD) of the log_10_ values.

## 4. Discussion

The pathogenesis of *C. perfringens* gas gangrene has been extensively studied since the pioneering work of the 1940s [42,43]. By using mouse models, much has been learned about how this bacterium causes clostridial myonecrosis [3,5,9,10,28,31]. However, those previous studies have used only wild-type mice, often the BALB/c strain. Consequently, one important aspect of gas gangrene has received little research attention, i.e., why is diabetes mellitus such an important host risk factor for *C. perfringens* gas gangrene [15,16,18–21].

To begin addressing this question, we established a *db/db* mouse model for assessing host type 2 diabetes contributions to the development of *C. perfringens* gas gangrene. Those *db/db* mice are derived from C57BL/6J WT mice, so we compared the effects of a *C. perfringens* type A strain ATCC3624 challenge of mouse thigh muscle challenge for *db/db vs*. C57BL/6J WT mice. ATCC3624 is representative of the type A strains that cause gas gangrene and has been employed previously for *C. perfringens* gas gangrene studies using BALB/c mice [4]. Our first observation was that, by comparing results of our previous study [4] with the current results, WT C57BL/6J mice appear to be less affected than BALB/c mice by a 10^6^ intra-thigh challenge with ATCC3624, i.e., there appear to be differences in gas gangrene susceptibility even among WT mouse strains. The genetic basis for such WT mouse strain variations in developing gas gangrene requires further study.

When *db/db* and WT C57BL/6J mice were similarly challenged for 4 h with ATCC3624, both strains of mice developed gas gangrene. However, at this timepoint, the *db/db* mice presented more severe clinical signs, and gross and microscopic lesions, compared to their WT counterparts. This increased sensitivity suggests *db/db* mice are useful as an animal model for probing the strong association between *C. perfringens* gas gangrene and type 2 diabetes. Notably, this determination appears to be novel since a literature search identified only 1 previous *C. perfringens* paper using diabetic mice [44] to examine polymicrobial abscess formation but did not identify any previous publications describing *C. perfringens* gas gangrene in diabetic animal models. Related to this, gas gangrene in diabetics can also be polymicrobial [30] and the *db/db* diabetic mouse model of gas gangrene should also be useful for studying mixed infections with *C. perfringens* and other bacteria, where high glucose and BCAA levels in these diabetic mice might influence behavior of the overall microbial community as well as *C. perfringens*.

The current study utilized a relatively early (4 h) infection of *db/db* mice for ethical reasons, i.e., to reduce pain and suffering. This model should also be extendable to a longer challenge time, which would allow investigation of parameters such as survival (no animals died in the current 4 h challenge) or biofilm formation on necrotic muscle (note that *C. perfringens* biofilm formation is generally studied at times longer than 4 h) [45,46]). However, an ethical issue that would need to be considered for longer challenges is pain and suffering, especially since use of opioid analgesics is not possible for these mice since they significantly interfere with the development of gas gangrene in mice [47].

Our initial work also provides some suggestions regarding why the *db/db* mice developed more severe lesions than their wild-type parents when challenged for 4 h with equivalent *C. perfringens* doses. This effect apparently does not involve more growth of ATCC3624 in the *db/db* mice *vs*. WT C57BL/6J mice under the experimental conditions used; i. e., at the 4 h conclusion of this study, no consistent difference in either total or viable ATCC3624 numbers were detected in the left thigh of *db/db vs*. WT mice at any challenge dose. If ethical issues can be overcome (see above), a time course study comparing infection in *db/db* vs. WT mice for longer challenge periods should be performed in the future since it likely takes time for *C. perfringens* injected into muscle to produce sufficient toxin levels to cause necrosis and create favorable low tissue redox conditions, and perhaps help liberate nutrients, to foster significant increases in *C. perfringens* numbers in muscle. Therefore, with longer challenge times, it is possible that more favorable redox conditions generated by toxins may result in detectable differences in *C. perfringens* growth between the *db/db* and WT mice.

The *db/db* mouse is considered, at least in part, a good model for type 2 diabetes [32,48,49] because, like type 2 diabetic patients [22], these mice develop high blood levels of glucose and branched chain amino acids (BCAAs) and become hypoinsulinemic [5,6,19,22,26, https://www.jax.org/strain/000642]. Before challenging mice with ATCC3624, we confirmed that the *db/db* mice had much higher levels of glucose and BCAAs compared to WT C57BL/6J mice. It is possible that these metabolic alterations contribute to the more severe lesions observed in the *db/db vs* WT mice since elevated BCAA levels are thought to impact glucose metabolism in diabetics and may suppress their wound healing and immune responses [24,50]. Therefore, another future study might compare immune responses, such as cytokine levels, between *db/db* vs WT mice after *C. perfringens* challenge.

*C. perfringens* cannot make BCAAs and must obtain BCAAs from the host for producing toxins during disease [51], so it is also possible that high blood BCAA levels in diabetics favors increased toxin production. This possibility raises an intriguing question from our results; why do the *db/db* and WT mice have a similar bacterial load in their challenged muscle, yet the clinical signs and lesions are more severe in *db/db* than in WT mice? To start investigating a possible mechanism behind these findings, we performed RT-qPCR analysis for *cpa* and *pfo*A expression on muscle from infected animals, as a proxy for toxin production in those tissues since our previous attempts to reliably detect the production of PFO and CPA in the presence of muscle cells were unsuccessful [12], as also experienced using infected muscle during the current study (not shown). At 2 h post-challenge with *C. perfringens*, RT-qPCR detected significantly stronger expression of the genes encoding CPA and PFO, which are the toxins mediating gas gangrene [3,5], in the *db/db* vs. the WT mice. Toxin gene expression was measured at 2 h based upon, i) our previous *in vitro* infection studies [12] showing that, in the presence of C2C12 differentiated mouse muscle cells, ATCC3624 upregulates expression of genes encoding CPA and PFO within 2 h and ii) the lack of any growth of ATCC3624 even up to 4 h after infection in either the *db/db* or WT mice. Given the moderate correlation between bacterial gene expression levels and protein production levels [52], these toxin gene expression results suggest that more CPA and PFO may be produced in *db/db* vs WT mice early during gas gangrene infection. Such a difference would be relevant to disease severity since CPA and PFO are the main virulence factors of *C. perfringens* type A in cases of gas gangrene [3,5] and these two toxins can directly kill differentiated muscle cells *in vitro* and, by implication, muscle cells *in vivo* [12]. Therefore, these results suggest that the presence of a higher concentration of both toxins in the thigh muscles of *db/db vs* WT mice might help to explain the higher score of clinical signs and lesions in *db/db* mice in our studies.

## Data Availability

Data will be made available on request.
